# Transforming growth factor β‐mediated micromechanics modulates disease progression in primary myelofibrosis

**DOI:** 10.1111/jcmm.15526

**Published:** 2020-09-05

**Authors:** Patric Teodorescu, Sergiu Pasca, Ancuta Jurj, Grigore Gafencu, Jon‐Petur Joelsson, Sonia Selicean, Cristian Moldovan, Raluca Munteanu, Anca Onaciu, Adrian‐Bogdan Tigu, Mihail Buse, Alina‐Andreea Zimta, Rares Stiufiuc, Bobe Petrushev, Minodora Desmirean, Delia Dima, Cristina Vlad, Jon Thor Bergthorsson, Cristian Berce, Stefan Ciurea, Gabriel Ghiaur, Ciprian Tomuleasa

**Affiliations:** ^1^ Department of Hematology Iuliu Hatieganu University of Medicine and Pharmacy Cluj Napoca Romania; ^2^ Department of Hematology Ion Chiricuta Clinical Cancer Center Cluj Napoca Romania; ^3^ Research Center for Functional Genomics and Translational Medicine Iuliu Hatieganu University of Medicine and Pharmacy Cluj Napoca Romania; ^4^ Molecular Haematology Unit ‐ Weatherall Institute of Molecular Medicine John Radcliffe Hospital University of Oxford Oxford UK; ^5^ Stem Cell Research Unit, Biomedical Center School of Health Sciences University of Iceland Reykjavík Iceland; ^6^ Department of Laboratory Hematology Landspitali University Hospital Reykjavík Iceland; ^7^ Graduate School for Cellular and Biomedical Sciences Universität Bern Bern Switzerland; ^8^ Medfuture Research Center for Advanced Medicine Iuliu Hatieganu University of Medicine and Pharmacy Cluj Napoca Romania; ^9^ Department of Pathology Constantin Papilian Military Hospital Cluj Napoca Romania; ^10^ Department of Hematology Ion Chiricuta Clinical Research Center Cluj Napoca Romania; ^11^ Department of Cardiology Iuliu Hatieganu University of Medicine and Pharmacy Cluj Napoca Romania; ^12^ Department of Cardiology Rehabilitation Hospital Cluj Napoca Romania; ^13^ Department of Laboratory Hematology Landspitali University Hospital Reykjavík Iceland; ^14^ Animal Facility Iuliu Hatieganu University of Medicine and Pharmacy Cluj Napoca Romania; ^15^ Department of Cellular Therapies and Stem Cell Transplantation University of Texas MD Anderson Cancer Center Houston TX USA; ^16^ Department of Leukemia Sidney Kimmel Cancer Center at Johns Hopkins Johns Hopkins University School of Medicine Baltimore MD USA

**Keywords:** fibroblast activation, invasion, micromechanics, myelofibrosis, proliferation, TGF‐β

## Abstract

Primary myelofibrosis (PMF) is a Ph‐negative myeloproliferative neoplasm (MPN), characterized by advanced bone marrow fibrosis and extramedullary haematopoiesis. The bone marrow fibrosis results from excessive proliferation of fibroblasts that are influenced by several cytokines in the microenvironment, of which transforming growth factor‐β (TGF‐β) is the most important. Micromechanics related to the niche has not yet been elucidated. In this study, we hypothesized that mechanical stress modulates TGF‐β signalling leading to further activation and subsequent proliferation and invasion of bone marrow fibroblasts, thus showing the important role of micromechanics in the development and progression of PMF, both in the bone marrow and in extramedullary sites. Using three PMF‐derived fibroblast cell lines and transforming growth factor‐β receptor (TGFBR) 1 and 2 knock‐down PMF‐derived fibroblasts, we showed that mechanical stress does stimulate the collagen synthesis by the fibroblasts in patients with myelofibrosis, through the TGFBR1, which however seems to be activated through alternative pathways, other than TGFBR2.

## 1 INTRODUCTION

Primary myelofibrosis (PMF) is a Ph‐negative myeloproliferative neoplasm (MPN), characterized by advanced bone marrow fibrosis and extramedullary haematopoiesis that is most pronounced in the liver and spleen.[1] Among MPNs, approximately 50% of patients with PMF and essential thrombocythemia (ET) are *JAK2* V617F positive, whereas >90% polycythemia vera (PV) patients are positive for this mutation.[2, 3, 4] To date, several ‘driver’ mutations have been identified in the pathogenesis of PMF, involving target genes such as JAK2, CALR and MPL, whereas less commonly involved genes include ASXL1, SRSF1 and U2AF1.[5, 6, 7, 8] The bone marrow fibrosis results from proliferation of fibroblasts that are influenced by several cytokines in the microenvironment including transforming growth factor‐β (TGF β), basic fibroblast growth factor (FGF), platelet‐derived growth factor (PDGF) and calmodulin.[9, 10, 11, 12, 13] A likely source of these factors is the underlying malignant clone that may acquire different phenotypic states including megakaryocyte or monocyte differentiation.[12, 14, 15] In support of these data, we have recently shown that the fibroblasts involved in this process do not derive from the malignant clone but seem to represent over‐stimulated normal cells.[16]

The main cytokine believed to be involved in generation of PMF‐associated fibrosis is TGF β.[17] TGF‐β acts on fibroblast physiology by increasing synthesis of collagen, type I, III, IV and V, as well as production of fibronectin, proteoglycans and tenascin.[18] Although the importance of TGF β in fibroblast proliferation was previously underlined in studies of PMF, micromechanics related to the niche has yet been elucidated, as progression in PMF occurs in a tense niche, with a relatively high level of mechanical stress.[18, 19] The role of bone marrow mechanical stress via TGF‐β modulation was assessed by Balooch et al, with potential clinical implications.[20] Data were confirmed by Zhao et al[21] that have shown that bone marrow‐derived mesenchymal stem cells have resistance to flow shear stress through AMP‐activated protein kinase signalling.

In the current study, we hypothesized that mechanical stress produced during PMF progression modulates TGF‐β‐dependent signalling. TGF‐β is secreted by the malignant megakaryocytes, leading to further activation and subsequent proliferation and invasion of bone marrow fibroblasts, both in the bone marrow and in distant organs. Thus, we demonstrate the important role of micromechanics in the microenvironment and the development and progression of PMF.

## 2 MATERIALS AND METHODS

### 2.1 Cell cultures

**Figure 1:**
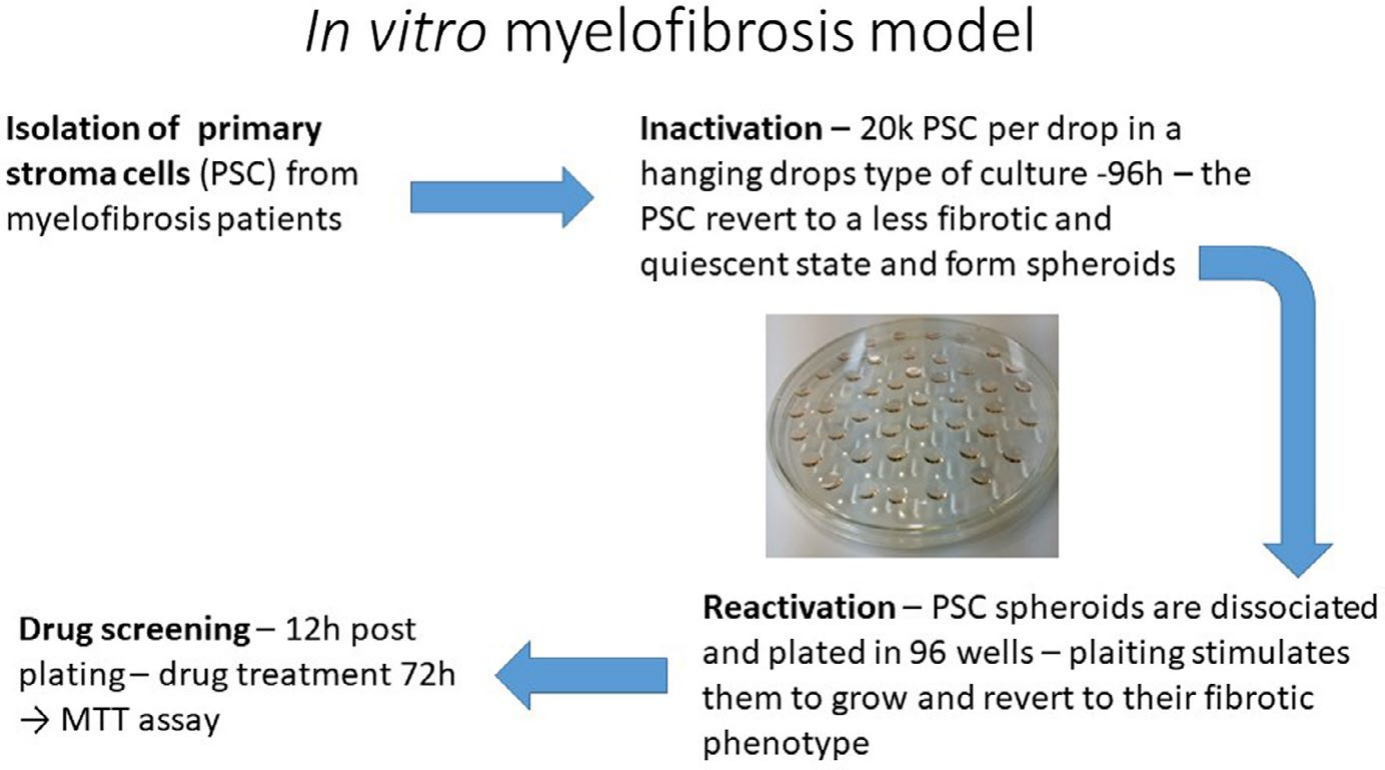
Isolation and characterization of PMF‐derived fibroblasts

Primary myelofibrosis‐derived fibroblast cell lines used were isolated and characterized as previously published [16] (Figure 1). All cells were grown in a humidified atmosphere at 37°C air, 95%; carbon dioxide (CO_2_), 5%. Cell passage and cultures were carried out as previously described.[22, 23, 24] All cells were cultured in Dulbecco's modified Eagle's medium (DMEM), supplemented with penicillin/streptomycin and non‐essential amino acids. Hanging drops were used to isolate the cells from any external stimuli, and cells were further treated in hanging drops for 4 days until spheroids were formed and the fibroblasts were inactivated. Re‐activation of the fibroblasts was carried out by digesting the spheroids with trypsin and culture on a plastic dish. Once re‐activated, proliferation and collagen type I and III increased. For the hanging drop experiments, classic Petri dishes were used, making this experiment feasible and reproducible in any laboratory setting. Images of the cells were obtained by inverted phase microscopy from activated, re‐activated as well as organoids in a 30‐μ dish (50 mm) using an inverted Zeiss Axio Observer Z1 microscope, as previously described.[25, 26, 27]

### 2.2 shRNA TGFBR1 and TGFBR2 knock‐down

Transforming growth factor‐β receptor (TGFBR) 1 and 2 knock‐down PMF‐derived fibroblasts were obtained in collaboration with the Department of Oncology, the Johns Hopkins University School of Medicine in Baltimore, USA. Briefly, cells were plated at density of 5 × 10^6^ cells/plate confluence in 8 mL of DMEM with 10% FBS with no penicillin/streptomycin. At Day 2, cells were transfected with lipofectamine. The DNA mix was shRNA plasmid (9⎧g) + delta8.9 (9⎧g) + VSVG (3⎧g) + 800 µL Opti‐MEM. Medium was changed the following day. Virus titre was at its highest approximately 24 hours after transfection. The medium was passed through a 0.45 µm filter unit attached to a syringe. Transduction of cells with lentivirus supernatant and selection with puromycin was carried out at Day 5. The puromycin selection process was done using 4‐4.25 µg/mL for PMF‐derived fibroblasts and continued until all non‐transduced control cells were dead. Later, living cells were transferred to T25 flasks and expanded before assessment of the knock‐down via qPCR.

### 2.3 Assessment of micromechanics on the PMF‐derived fibroblasts

Following TGFBR1 and TGFBR2 knock‐down, the role of mechanical pressure of the cells was assessed on three cell lines: TGFBR1 knock‐down cells, TGFBR2 knock‐down and wild‐type cells, used as negative control. Micromechanics was assessed using the Flexcell FX‐6000 Tension System, in collaboration with the Stem Cell Research Unit, Biomedical Centre, School of Health Sciences, University of Iceland—Department of Laboratory Hematology, Landspitali University Hospital, Reykjavík, Iceland, as previously described.[28, 29, 30, 31, 32] Briefly, cells were cultured as described above. Equal number of cells were seeded on each well in a 6‐well BioFlex plates (Flexcell International Corporation, Burlington, CA, USA) and grown to approximately 80% confluence. These plates were then transferred to a base plate of the cell stretching equipment Flexcell FX‐5000TM Tension System (Flexcell International Corporation) in a humidified incubator at 37°C and 5% CO_2_. The cells were subjected to cyclic mechanical stretch with the following parameters: a stretching rate of 20% with a square signal, 0.33 Hz frequency (20 cycles/min) and a 1:1 stretch: relaxation ratio. The cells were stretched for 6 and 24 hours as described in the results. Control BioFlex plates were kept in the same incubator under static conditions as non‐stretch controls. We compared fibroblasts obtained from myelofibrosis patients with a healthy bone marrow stroma, with the obtained data presented in the results section. A dashed line is used to illustrate a fold change of 1. Up‐regulation of TGFBR1 and down‐regulation of TGFBR2 were assessed in both primary lines (experimental groups) compared with normal stroma (control cells).

### 2.4 DNA and RNA extraction

Total RNA was isolated using TRIzol reagent (Invitrogen), as previously described.[33, 34, 35]

### 2.5 Quantitative RT‐PCR (q RT‐PCR) for mRNA expression

Quantitative RP‐PCR was performed to confirm the expression of the collagen type I and type III expression, using TaqMan PCR technology.[36, 37, 38] For the PCR, following first‐strand synthesis, 10 μL of RT reaction mixture was used for subsequent PCR amplification by adding 40 μL of PCR master mix to the same wells. The PCR reaction mixture included 1X PCR buffer, 200 μmol/L of each dNTP and 1.25 U Taq polymerase (Roche Molecular Biochemicals, Indianapolis, IN, USA) in a final volume of 50 μL. Amplification and real‐time data acquisition were performed in an ABI Prism 7700 Sequence Detector using the following cycle conditions: initial denaturation at 95°C for 1 minute, followed by 40 cycles of denaturation at 95°C for 12 seconds, and annealing at 60°C for 1 minute. The primers used for collagen I: the forward primer is 5'‐CATGACCGAGACGTGTGGAAACC‐3', and reverse primer is 5'‐CATGACCGAGACGTGTGGAAACC. The primers used for collagen III: the forward primer is 5'‐GGATCAGGCCAGTGGAAATGTAAAGA‐3', and reverse primer is 5'‐CTTGCGTGTTCGATATTCAAAGACTGTT. Cycle passing threshold (Ct) was recorded and normalized to human GAPDH (hGAPDH) expression. Relative expression was calculated as 2Ct_FLT3‐Ct_GAPDH. PCR reactions were carried out in triplicate. All mRNA q RT‐PCR values were normalized to β‐actin, and the relative expression was calculated as 2Ct_target gene‐Ct_GAPDH. PCR reactions were carried out in triplicate. As internal controls, we used hGAPDH forward, 5′‐GTGGTCTCCCTGACTTTCAACAGC‐3′, and hGAPDH reverse, 5′‐ATGAGGTCCACCACCTGCTTGCTG‐3′ (149‐bp amplicon).

### 2.6 Western blotting

Cells were lysed in Laemmli sample buffer (Bio‐Rad, Hercules, CA, USA) supplemented with a protease inhibitor complete EDTA‐free (Roche). Protein concentration was measured using BCA Protein Assay kit (Pierce, Rockford, MA, USA). Cell lysates (50 µg) were electrophoresed on 10%‐20% polyacrylamide gels (Bio‐Rad) and transferred to Immobilon PSQ membranes (Millipore, Bedford, MA, USA). The membranes were blocked with TBS containing 5% skim milk and 0.1% Tween‐20, then incubated with the primary antibody overnight, at 4°C. Antibodies for collagen I and collagen III were purchased from Abcam (Cambridge, UK). The membranes were incubated after washing with HRP‐conjugated goat anti‐rabbit IgG (Calbiochem, Gibbstown, NJ, USA) and analysed using enhanced chemiluminescence plus reagent (GE Healthcare, Buckinghamshire, UK).

### 2.7 Animals and housing for the in vivo experiments

Athymic nude mice Crl:NU(Ncr)‐Foxn1nu mice (Charles River, Laboratories, Wilmington, MS, USA) were used in the present study. The animals were housed in polysulfone type II‐L open‐top cages (Tecniplast Buguggiate, Italy) and had access to filtered tap water in bottles and pelleted feed (Cantacuzino Institute, Bucharest, Romania) ad libitum. The bedding was a standard wood chip aseptic bedding (Lignocel®; J. Rettenmaier & Söhne GmBH+Co. KG, Rosenberg, Germany). The mice were bred and kept in the Animal Facility of the Medfuture Research Center for Advanced Medicine, Iuliu Hatieganu University of Medicine and Pharmacy at a standard temperature of 22°C ± 2°C and a relative humidity of 55% ± 10%, in a 12:12‐hour light:dark cycle (lights on, 7 am to 7 pm) at a light intensity of 300 lx at 1 m above the floor, and were allocated into two groups: group A (experimental group) and group B (control group). The groups were randomized in different cages, and the individuals were numbered on the base of their tail using permanent makers (Sharpie, Oak Brook, IL, USA). All experimental protocols were approved by the Ethics Committee of Iuliu Hatieganu University of Medicine and Pharmacy and were conducted in accordance with the EU Directive 63/2010. Prior to the onset of the study, all animals were quarantined and left to acclimatize to the separation from the mice colony for 10 days. For environmental enrichment, autoclaved braided cotton dental rolls were used (Celluron®; Hartmann, Heidenhelm, Germany). The study was designed and performed in accordance with the ARRIVE Guidelines for Reporting Animal Research. All animal‐handling procedures were performed according to the European and Romanian guidelines.

Group A (experimental group) mice were injected with GFP‐positive PMF‐derived fibroblasts in the central venous system, whereas group B (control group) were injected with normal bone marrow‐derived fibroblasts. The in vivo experiments allowed us to investigate whether PMF‐derived fibroblasts had the potential to invade hematopoietic organs following injection in the systemic blood flow of immunocompromised animals. One month following injection of 1 × 10^6^ cells in the central venous system, all animals were sacrificed, and pathology slides were used to assess both the bone marrow and lung tissue using dark‐field microscopy.

### 2.8 Confocal microscopy

Confocal microscopy was performed on an Olympus FLUOVIEW FV1200 laser scanning confocal microscope (Olympus, Tokyo, Japan). Image acquisition was performed using the UPLSAPO 10X2 (0.4 NA) objective. The images were obtained using channel mode (488 nm excitation and bright field). Other settings for the image acquisition were determined using the FV10‐ASW 4.2 software. Images were processed using ImageJ.

### 2.9 Transthoracic echocardiography in PMF patients

Routine transthoracic echocardiography (TTE) (Phillips Affinity 50G echograph) was used to investigate pulmonary hypertension in PMF patients. TTE was carried out by a certified cardiologist, experienced in the clinical evaluation of patients with chronic cardiopulmonary diseases and evaluated the right ventricle/left ventricle basal diameter ratio, flattening of the interventricular septum, right ventricular outflow Doppler acceleration time, pulmonary artery diameter and inferior vena cava diameter.

### 2.10 Statistical analysis

The data were analysed using two statistical software packages (GraphPad Prism 5.0 and R; GraphPad software, Inc, La Jolla, CA, USA). Initial results were assessed by using the Shapiro‐Wilk test to determine whether the distribution was normal. The distribution of all the obtained data was Gaussian; thus, it was analysed using a parametric test (two‐way ANOVA with Tukey post‐test). The differences were considered significant when *P* value was <0.05.

## 3 RESULTS

### 3.1 Knock‐down of TGFBR1 and TGFBR2 in PMF‐derived fibroblasts

**Figure 2:**
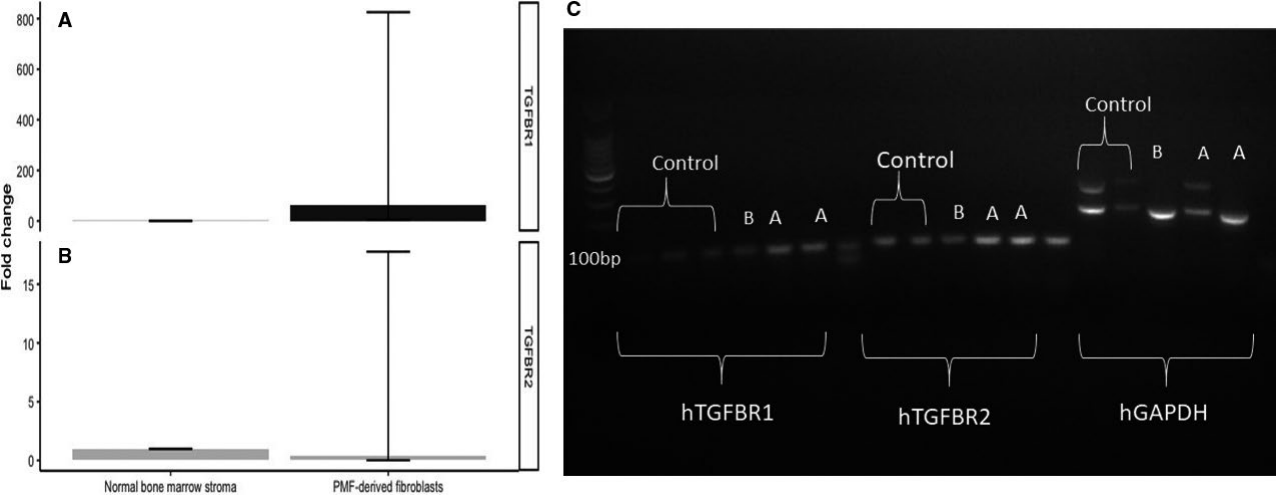
A, PCR analysis for the knock‐down of TGFBR1. B, PCR analysis for the knock‐down of TGFBR2. C, 2% agarose gel with TGFBR1 and TGFBR2 PCR products of the knock‐down PMF‐derived fibroblasts. Images A and B are the two PMF‐derived fibroblasts, whereas the control is represented by normal bone marrow fibroblasts

Four shRNA candidates were selected for TGFBR1 (TRCN0000039775 and TRCN0000039776) and TGFBR2 (TRCN0000000833 and TRCN0000000834). Lentiviral supernatants were subsequently generated carrying 3 types of shRNA (TRCN0000000834, TRCN0000039775 and TRCN0000039776). These were successfully transduced to and selected (4.5 μg/mL puromycin) for in PMF‐derived fibroblast cells with the following constructs: pGIPZ—scrambled shRNA, TRCN0000000834, TRCN0000039775 and TRCN0000039776, as shown in Figure 2A and B. The result of the knock‐down is shown in Figure 2C that depicts a 2% agarose gel with TGFBR1 and TGFBR2 PCR products of the knock‐down cells. pGipz depicts PMF‐derived fibroblasts with a blank vector, A cells depict PMF‐derived fibroblasts with TGFBR1 knock‐down, and B cells depict PMF‐derived fibroblasts with TGFBR2 knock‐down. The data show that we successfully integrated the shRNA and pGIPZ constructs in PMF‐derived fibroblasts, and from a transcriptomic point, the knock‐down for TGFBR2 was successful. Higher passage number can negatively impact transduction efficacy (data not shown), and the phenomenon observed for the TGFBR2 knock‐down might be a compensatory mechanism or the heterodimer nature of the receptor.[39]

### 3.2 Micromechanics of PMF‐derived fibroblasts

**Figure 3:**
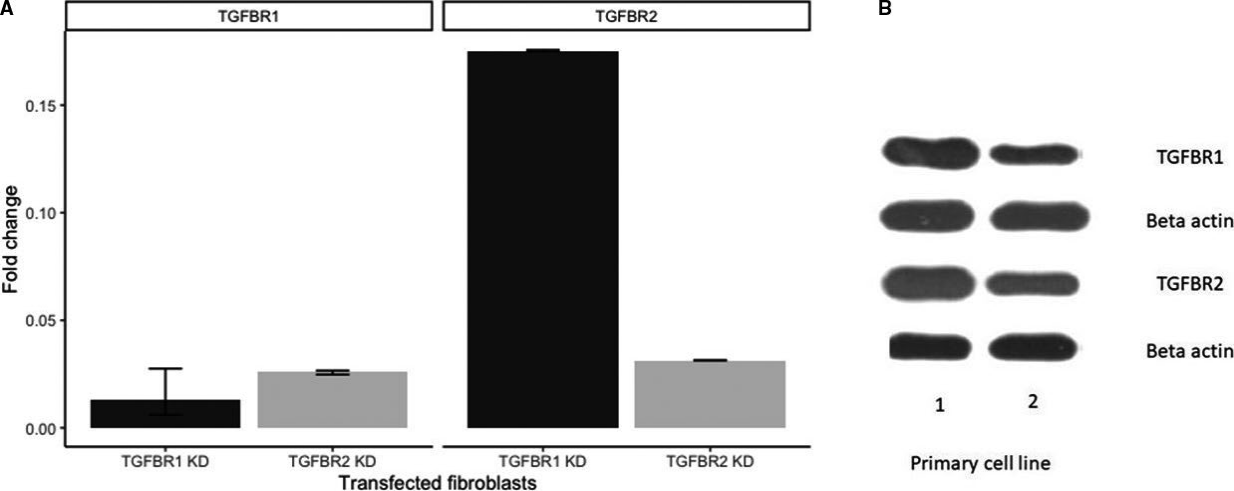
A, RT‐PCR shows that myelofibrosis‐derived fibroblasts, acting as cancer‐associated fibroblasts, are highly dependent on TGFBR1, with a possible compensatory down‐regulation of TGFBR2. B, Western blotting shows that myelofibrosis‐derived fibroblasts, acting as cancer‐associated fibroblasts, are highly dependent on TGFBR1, with a possible compensatory down‐regulation of TGFBR2

We compared fibroblasts obtained from myelofibrosis patients with a healthy bone marrow stroma. A dashed line is used to illustrate a fold change of 1. Up‐regulation of TGFBR1 and down‐regulation of TGFBR2 can be observed in both primary lines compared with normal stroma. This can lead to the hypothesis that myelofibrosis‐derived fibroblasts, acting as cancer‐associated fibroblasts, are highly dependent on TGFBR1, with a possible compensatory down‐regulation of TGFBR2 (Figure 3A). As shown in Figure 3B, Western blotting confirmed the RT‐PCR data.

**Figure 4:**
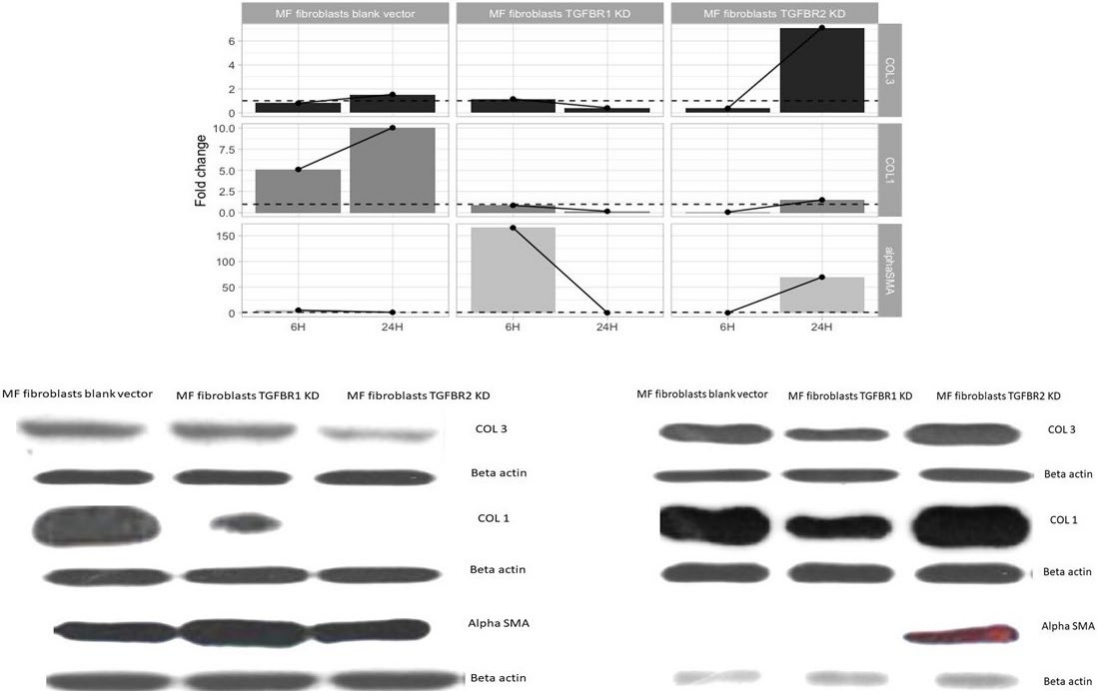
A, RT‐PCR shows the role of mechanical stress is shown on PMF‐derived fibroblasts. B, Western blotting data at 6 hours show the role of mechanical stress is shown on PMF‐derived fibroblasts. C, Western blotting data at 24 hours show the role of mechanical stress is shown on PMF‐derived fibroblasts

The influence of mechanical stress is shown in Figure 4A (RT‐PCR data), B (Western blotting data at 6 hours) and C (Western blotting data at 24 hours). Fold change was determined using the delta CT method. β2‐microglobulin was used as housekeeping gene. We compared fibroblasts under tension *versus* control fibroblasts; thus, a fold change under 1 was considered as down‐regulated in fibroblasts under tension compared with control fibroblasts and a fold change over 1 was considered as up‐regulation in fibroblasts under tension compared with control fibroblasts. The dashed line is used to illustrate a fold change of 1. Scales differ between gene expressions because fold changes vary widely between them, so we considered that a free *y*‐axis would be a more appropriate representation.

### 3.3 Invasive potential of PMF‐derived fibroblasts

**Figure 5:**
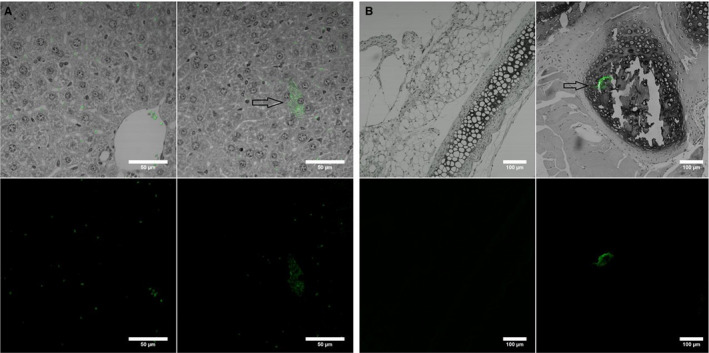
GFP‐positive PMF‐derived fibroblasts in the lungs (A) and bone marrow (B) of the nude mice using confocal microscopy

After proving that micromechanics plays a role in PMF progression following PMF‐derived fibroblast activation and increased proliferation, we aimed at assessing the potential of these cells, acting as cancer‐associated fibroblasts, to invade distant organs. Thus, we used nude immunocompromised mice for in vivo experiments. Following the injection of GFP‐positive PMF‐derived fibroblasts (green fibroblasts), as well as normal fibroblasts, acting as the control group, into the systemic blood flow of nude mice, we evaluated their invasive potential 4 weeks later. Animals were killed, and pathology slides were used to assess the internal organs and bone marrow of the mice. We identified GFP‐positive cells in the lungs (Figure 5A) and bone marrow (Figure 5B) of the nude mice using confocal microscopy. The upper images represent the bright‐field microscopy merged with the channel corresponding to GFP emission, and the lower images show only the green channel. In the right images, we have the experimental group (with GFP‐positive cells pointed using an arrow), whereas in the left images we have the control group, lacking any GFP‐positive cell. The autofluorescence of paraffin was removed using the FV10‐ASW 4.2 software, but the autofluorescence of red blood cells was still visible.

### 3.4 Clinical evaluation of pulmonary hypertension in PMF patients

**Figure 6:**
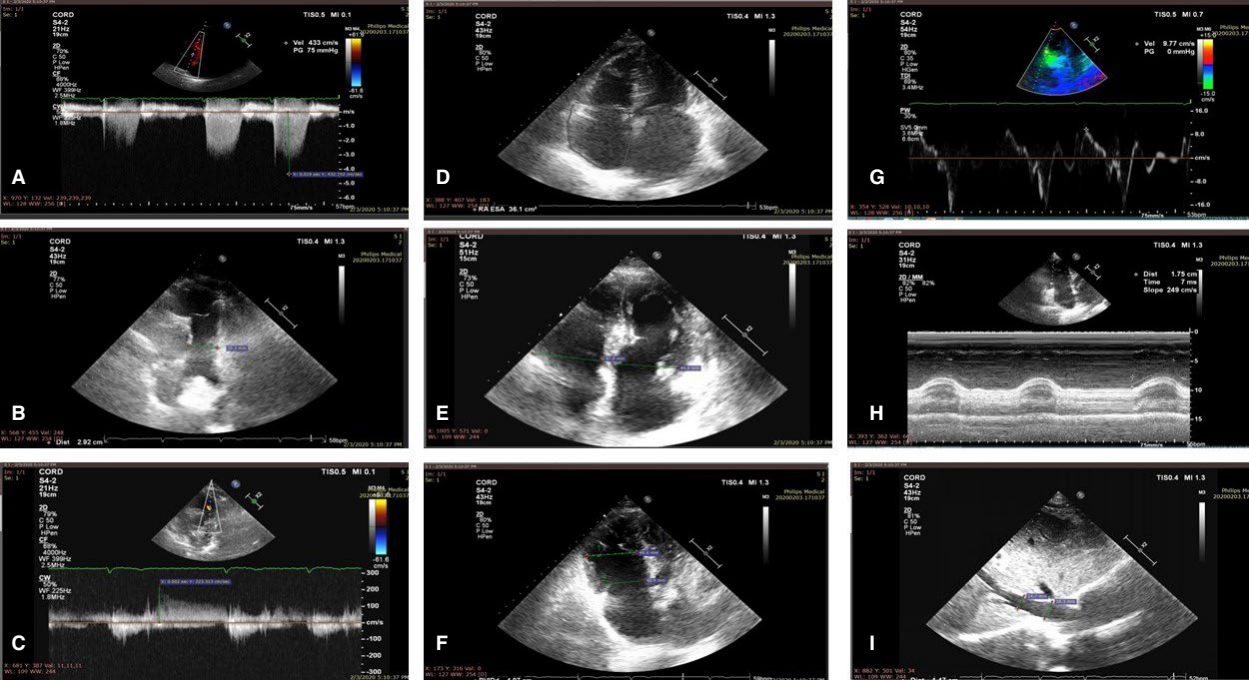
A‐I, Echography assessment of pulmonary hypertension in PMF

After showing that PMF‐derived fibroblasts have homing potential in the lung tissue, with possibly subsequent altering of the local mechanics, we assessed pulmonary hypertension in PMF patients by echocardiography (Figure 6A‐I). When assessing pulmonary hypertension in PMF patients, we used a small cohort of patients (n = 5) for a preliminary proof‐of‐concept study. The link between patient characteristics, data on age, disease status or comorbidities and development of pulmonary hypertension is elaborated in the discussions section of the article. Peak tricuspid velocity (TRV) is depicted by the apical 4‐chamber (A4C) view—CW Doppler. The peak velocity is 4.3 m/s. A TRV > 2.8 m/s is considered abnormal (Figure 6A). Pulmonary artery (PA) diameter is shown in Figure 6B, with a parasternal short‐axis (PSAX) view—2D image. PA dimension is measured in end‐diastole halfway between the pulmonary valve and bifurcation of main PA. PA diameter is 29 mm. A diameter of >25 mm is considered abnormal. Figure 6C depicts the early diastolic PR velocity PSAX‐CW Doppler measurement through the pulmonary valve in line with the PR jet. The peak (early/beginning of diastole) PR velocity value is measured. An early PR velocity >2.2 m/s is considered a marker of raised mean PAP. Right atrial (RA) area is presented in Figure 6D. The RA from the plane of the tricuspid valve annulus along the interatrial septum, superior and lateral walls of RA. RA area is 36 cm2. RA area >18 cm^2^ is considered abnormal, a sign of pulmonary hypertension. RV/LV basal diameter ratio is presented in Figure 6E. This is measured from the standard A4C view without foreshortening. Measurement is taken at end‐diastole. Ratio of >1 measured at end‐diastole suggests RV dilatation (47.9/44.9). Figure 6F presents right ventricle (RV) dimensions. Basal RV diameter is measured at the maximal transverse diameter in the basal one third of the RV. RVD2 > 41 mm is abnormal. RVD1: Mid RV diameter is measured at the level of the LV papillary muscles. RVD1 > 35 mm is abnormal. Figure 6G presents the RV pulsed tissue Doppler S wave (Sʹ) velocity. PW tissue Doppler S wave measurement is taken at the lateral tricuspid annulus in systole. Sʹ wave velocity <9.5 cm/s indicates RV systolic dysfunction. Figure 6H presents the tricuspid annular plane systolic excursion (TAPSE). The excursion of the lateral tricuspid annulus is measured by M‐mode between end‐diastole and peak systole for a measure of longitudinal RV systolic function. TAPSE < 1.7 cm is highly suggestive of RV systolic dysfunction. Finally, Figure 6I shows the inferior vena cava diameter (IVC) Subcostal (2D M‐mode). The diameter is measured perpendicular to the IVC long axis, 1 cm from the RA junction at end expiration. The IVC diameter >21 mm with decreased inspiratory collapse (<50% with a sniff or <20% with quiet respiration) is considered abnormal.

## 4 DISCUSSIONS

**Figure 7:**
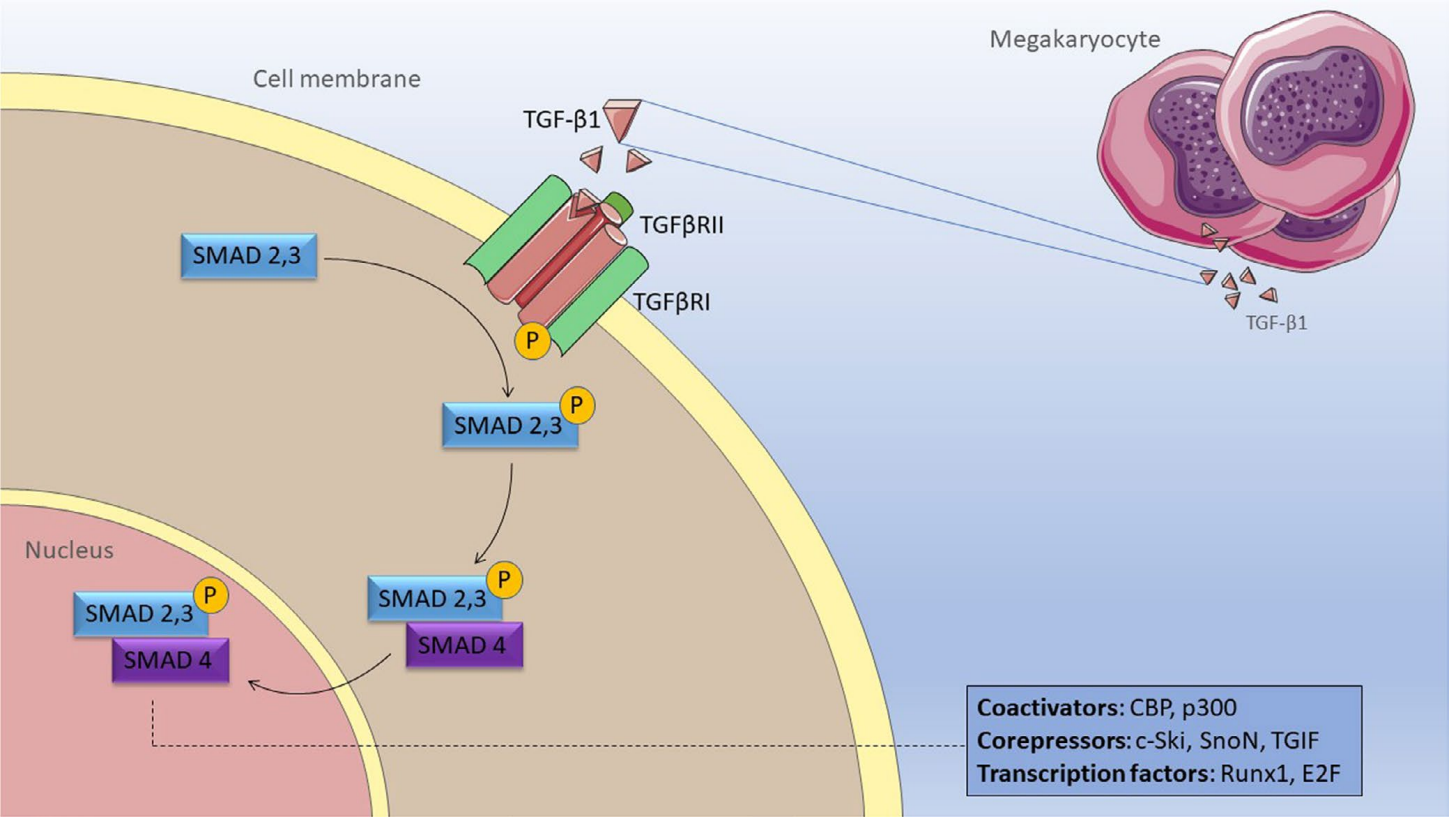
Molecular mechanism of TGF‐beta receptor action

TGF‐β plays a major role in the biology of primary myelofibrosis, is being secreted by the malignant megakaryocytes and is likely further affecting the bone marrow microenvironment.[12, 40] TGF‐β is a cytokine secreted in 3 isoforms: TGF‐β1, TGF‐β2 and TGF‐β3, out of which TGF‐β1 is the most abundant.[41, 42] TGF‐β1 is initially secreted in a latent form and further activated by reactive oxygen species, as well as various proteases such as plasmin, integrins and trombospondin‐1, and binds to two types of receptors: type I (TGFβR1) and type II (TGFβR2).[43] Initially, TGF‐β1 binds to TGFβR2; then, TGFβR2 stimulates the protein kinase activity of TGFβR1 by recruiting, binding and transphosphorylating it[44] (Figure 7). Further on, the transcription factors Smad2/3 are recruited and phosphorylated, which bind next to Smad4 and translocate in the nucleus where they interact with coactivators (as are CBP and p300), co‐repressors (as are c‐Ski, SnoN or TGIF) and transcription factors (as are Runx1 or E2F), thus regulating the transcription of TGF‐β‐responsive genes.[44] Smad phosphorylation was linked to bone marrow physiology by Lee et al,[45] who showed that BMP signalling through both Smad and p38 mitogen‐activated protein kinase (MAPK) modulates the differentiation of mesenchymal stem cells in the bone marrow microenvironment. Cell shape and cytoskeleton micromechanics are altered by Smad protein phosphorylation in the bone marrow embryology via RhoA/ROCK‐mediated tension generated by BMP‐induced signalling.[46] Other groups have looked at the essential interplay between biochemical and mechanical cues in cell differentiation and as micromechanics plays key roles in both the normal physiology and embryology.[47] Our hypothesis was that mechanical stress might modulate the progression of primary myelofibrosis by targeting TGF‐β pathways in the bone marrow microenvironment.

Our results show an increase in collagen III, collagen I and alpha‐SMA fold change at 24 hours when compared to 6 hours in PMF‐derived fibroblasts blank vector. The same is seen in MF fibroblasts TGFBR2 KD (except for alpha‐SMA expression in PMF fibroblasts blank vector). In contrast, PMF fibroblasts TGFBR1 KD presented a decrease in COL3, COL1 and alpha‐SMA fold change at 24 hours compared to 6 hours, showing the importance of TGFBR1, but not TGFBR2 in mechanical stress signalling in PMF fibroblasts. A possible explanation for these findings could be the exhaustion of fibroblasts when TGFBR1 signalling is down‐regulated. It should be mentioned that this effect takes place although a cross‐reactivity between TGFBR1 and TGFBR2 has been observed, although not at the same magnitude. Another point to take into consideration is the higher starting expression of TGFBR1 compared with TGFBR2 in the primary fibroblasts, which can be observed further down the line in fibroblasts transfected with blank vector (data not shown). In conclusion, TGFBR1 has a greater drop in expression considering absolute values compared with TGFBR2, which argues in favour of the importance of TGFBR1 in mechanical stress response.

We also assessed collagen I and III secretion for the TGFβR1 knock‐down cells and found an important decrease in synthesis, both at the 6 and 24 hours following the exposure to micromechanical stress. Interestingly, the secretion of both collagen I and III at 24 hours was lower than the one at 6 hours, which suggests that the mechanism of synthesis was exhausted over time. In addition, the secretion of alpha‐SMA was higher in TGF‐β.

R1 knock‐down cells at 6 hours, data not confirmed in any of the other cell lines, which suggests that the loss of TGFβR1 enabled a mechanism of secretion for alpha‐SMA triggered by mechanical stress. However, this mechanism also was exhausted after 24 hours, as it was previously shown that the secretion of alpha‐SMA decreased close to zero.[48, 49, 50] For TGFBR2 knock‐down cells, collagen I and III were less expressed at 6 hours, in comparison with the wild‐type cells. However, the secretion of collagen I at 24 hours had the highest level of the 3 cell lines. Comparing the data from all 3 cell lines, if mechanical stress stimulates fibroblasts through this pathway, it suggests the activation of the TGFBR1 occurs through an alternative, more potent co‐receptor. The secretion of alpha‐SMA was close to undetectable at 6 hours but increased at 24 hours.

Cancer‐associated fibroblasts are known and described to promote tumour progression and invasion but are reported to lack any potential to invade surrounding tissues themselves.[51, 52, 53, 54] Still, our results show that PMF‐derived fibroblasts, acting as cancer‐associated fibroblasts, if brought to the systemic blood flow, have the ability of ‘homing’ in the bone marrow and extramedullary site of haematopoiesis, including the lung. Lefrancais et al have shown that the lung is a site for platelet biogenesis and haematopoiesis.[55] We show that such fibroblasts, once they migrate into the hematopoietic organ, may it be the bone marrow or an extramedullary site, may be activated and local mechanical pressure increases, thus promoting PMF. We indeed showed that micromechanics is altered in the bone marrow, not bringing forward any evidence for the same process occurring in the lung tissue. Still, clinical data have shown that PMF patients do indeed experience pulmonary hypertension,[56, 57, 58] as did our patient cohort, according to international echocardiography guidelines.[59]

When assessing pulmonary hypertension in PMF patients, we used a small cohort as a proof‐of‐concept preliminary study. Still, the link between hypertension in the lung parenchyma and PMF was just published in April 2020 by the Lopez‐Mattei and Verstovsek et al, using a 143 cohort of PMF patients.[60] They used echocardiogram and proved that 14% of patients had echocardiographic findings consistent with PH. Older age, male gender, hypertension, hyperlipidemia, coronary artery disease, dyspnoea, haematocrit, brain natriuretic peptide (BNP) and N‐terminal prohormone BNP (NT‐pro‐BNP) were significantly different between those without and those with pulmonary hypertension (*P* < 0.05). NT‐pro‐BNP was a significant clinical predictor of hypertension of the lung parenchyma (*P* = 0.006). Pulmonary hypertension in MF is lower than previously hypothesized, but many patients had cardiac comorbidities.

Even if further studies, both in the pre‐clinical setting and in the clinical data, must validate or invalidate our statement, we hypothesize that PMF‐derived fibroblasts have the potential to migrate in the lung parenchyma and change the local microenvironment, altering with the local micromechanics in order to promote disease progression and promote extramedullary haematopoiesis.

A major weakness of our study is that we used only a single‐cell in vitro analysis system—the PMF‐derived fibroblasts. These fibroblasts are not part of the neoplastic clone and are stimulated by malignant myeloid progenitors to proliferate and produce extracellular matrix, proven by Fialkow et al,[61] by Ciurea et al,[12] and by our group recently.[16] Thus, one might consider that the influence of mechanical stress on collagen synthesis might be linked to general fibroblast biology, rather than PMF. The same micromechanical stress might also act on malignant myeloid progenitors and their cytoplasmic protrusions that extend to the bone marrow. PMF basic biology should thus be investigated by using at least a co‐culture system of fibroblasts and their interaction with hematopoietic progenitors. The co‐culture is the next step in our research, as we aimed to understand each individual cell in the bone marrow microenvironment, this manuscript being of interest especially from a basic fibroblast biology standpoint.

## 5 CONCLUSION

In conclusion, our data suggest that mechanical stress does indeed stimulate the collagen synthesis by the fibroblasts in patients with myelofibrosis. Further studies are nevertheless needed to elucidate the exact pathway or pathways through which it acts, as our studies suggest that it is the mechanism does not follow the TGF‐β pathway completely and other downstream pathways should be investigated in the future, such as MAPK, ROCK or retinoic acid‐mediated pathways.

## CONFLICT OF INTEREST

There is no conflict of interest to be declared.

## AUTHOR CONTRIBUTION

**Patric Teodorescu:** Conceptualization (equal); Data curation (equal). **Sergiu Pasca:** Conceptualization (equal); Data curation (equal); Formal analysis (equal). **Ancuta Jurj:** Formal analysis (equal); Investigation (equal); Methodology (equal). **Grigore Gafencu:** Investigation (equal); Methodology (equal). **Jon‐Petur Joelsson:** Data curation (equal). **Sonia Selicean:** Formal analysis (equal). **Cristian Moldovan:** Data curation (equal). **Raluca Munteanu:** Methodology (equal). **Anca Onaciu:** Methodology (equal). **Adrian‐Bogdan Tigu:** Investigation (equal). **Mihail Buse:** Formal analysis (equal). **Alina‐Andreea Zimta:** Investigation (equal). **Rares Stiufiuc:** Investigation (equal). **Bobe Petrushev:** Formal analysis (equal). **Minodora Desmirean:** Formal analysis (equal). **Delia Dima:** Methodology (equal). **Cristina Vlad:** Formal analysis (equal). **Jon‐Thor Bergthorsson:** Methodology (equal). **Cristian Berce:** Investigation (equal). **Stefan Octavian Ciurea:** Conceptualization (equal). **Gabriel Ghiaur:** Conceptualization (equal); Supervision (equal). **Ciprian Tomuleasa:** Conceptualization (equal); Project administration (equal); Supervision (equal); Writing‐review & editing (equal).

## ETHICAL STATEMENT

The described experiments were carried out in accordance with all legal and ethical legislation requirements of Romania and the UE, in accordance with the Declaration of Helsinki. The in vitro experiments were carried out after the approval of the Ethics Committee of the Iuliu Hatieganu University of Medicine and Pharmacy in Cluj Napoca. Written informed consent was obtained from the individual for the publication of any potentially identifiable images or data included in this article.

## Data Availability

The data that support the findings of the study are available from the corresponding author upon reasonable request.
